# *Lycorisinsularis* (Amaryllidaceae), a new species from eastern China revealed by morphological and molecular evidence

**DOI:** 10.3897/phytokeys.206.90720

**Published:** 2022-09-19

**Authors:** Si-Yu Zhang, Hao-Tian Wang, Ying-Feng Hu, Wei Zhang, Song Hu, Jian-Wen Shao

**Affiliations:** 1 College of Life Sciences, Anhui Normal University, Wuhu, Anhui 241000, China; 2 College of Life Sciences, Anqing Normal University, Anqing, Anhui 246052, China; 3 Chengdu Shanhualangmanshi Gardening Limited Company, Chengdu, Sichuan 610000, China; 4 The Key Laboratory of Conservation and Employment of Biological Resources of Anhui, Anhui Normal University, Wuhu, Anhui 241000, China

**Keywords:** *Lycorissprengeri*, taxonomy, chloroplast genome, cryptic species

## Abstract

*Lycorisinsularis* S.Y.Zhang & J.W.Shao, a new fertile diploid species from coastal provinces in eastern China is described. This new species is most similar to *L.sprengeri* in morphology and has been misidentified as the latter for a long time. However, it can be distinguished from the latter by the relatively longer perianth tube (1.5‒2.5 cm vs. less than 1.3 cm), a characteristic that was overlooked before. Phylogenetic analysis, based on complete plastid genome, showed that *L.insularis* is not genetically related to *L.sprengeri* in the genus. The former was a sister group of *L.sanguinea*, while the latter was closely related to *L.longituba* and *L.chinensis* and they were respectively located on different clades that were separated at the base of the phylogenetic tree. The chromosome number of *L.insularis* is 2n = 22. At present, as the new species is relatively widely distributed and the wild population can normally reproduce by seeds, we evaluate it as LC (Least Concern) according to criteria of the IUCN Red List.

## ﻿Introduction

*Lycoris* Herb. (Amaryllidaceae) is a typical pan-East Asian genus, currently considered to have about 9 fertile diploid species, mainly distributed in China, Japan and South Korea (Hsu et al. 1994; Ji and Meerow 2000; Kim 2004; Hori et al. 2006; Lu et al. 2020; Zhang et al. 2021; Zhang et al. 2022). China is the centre of the distribution diversity of this genus, with about eight of these fertile diploid species being endemic to this country (Hsu et al. 1994; Lu et al. 2020; Zhang et al. 2021; Liu et al. 2022). Due to the special living habit of flowers and leaves not being able to co-exist, as well as the rich and beautiful flower patterns and colours, *Lycoris* plants are considered to have great horticultural value and have received extensive attention (Hsu et al. 1994; Zhang et al. 2022).

*Lycorissprengeri* Comes ex Baker was first described in 1902, being only distributed in China. The type specimen was collected from around Xiangyang City, Hubei Province in Central China (Comes and Baker 1902; Traub and Moldenke 1949; Hsu et al. 1994). Due to extensive investigation of basic plant resources in China, many populations identified as *L.sprengeri* were found and most of them were located in the coastal areas of Zhejiang, Fujian and Jiangsu Provinces. However, there were few collections or photographic records of this species in the central or inland areas near the collection location of the type specimens.

Recently, we collected some populations of *L.sprengeri* from inland areas, including Guangshui City, Hubei Province (near the collection location of the type specimen) and the surrounding areas of Dabie Mountains in Anhui Province. In the field, we noticed that there was a certain difference in morphology between those plants (identified as *L.sprengeri*) occurring inland and those from the coastal area: the flower colour of the former varied from white to dark purple in a population and the length of the perianth tube is short (less than 1.3 cm), while the flower colour of the latter is relatively stable, generally pink and the perianth tube is long (usually about 2.0 cm). After further morphological observations, chloroplast sequence alignment and phylogenetic analysis and checking the related type specimens, we recognised that the plants found in the inland area were the genuine *L.sprengeri*, while those plants collected from the coastal area (identified as *L.sprengeri*) are actually an undescribed new species. Thus, we named it as *Lycorisinsularis* S.Y.Zhang & J.W.Shao and describe it here.

## Materials and methods

### Plant collection and karyotype observation

Bulbs for study and observation were successively collected from the wild in 2016 to 2021. The information for the collection sites is shown in Table 1. Bulbs were cultivated in the Homogeneous Garden in Fengyang County, Chuzhou City, Anhui Province for continuous observation. In June 2022, bulb roots of 3‒5 individuals in each *Lycorisinsularis* population were induced by burying them in wet sand and the chromosome number was observed using the methods described by Chen and Li (1985). In August 2022, 50 flowers were randomly selected from each population for dissection and perianth tube lengths were measured. All statistical analyses were performed in SPSS ver. 19.0.

**Table 1.** Information of sampled populations and uploaded genomes.

Code	Locations	Altitude	GenBank acc. no
*L.sprengeri* (inland area)			
01	Yudian Town, Guangshui City, Suizhou City, Hubei Province	278 m	OP034616
02	Qiaotou Town, Mingguang City, Chuzhou City, Anhui Province	91 m	OP034617
03	Yinjian Town, Fengyang County, Chuzhou City, Anhui Province	137 m	OP034618
04	Huangjia Town, Tongcheng City, Anqing City, Anhui Province	122 m	OP034619
05	Pingshan Town, Huaining County, Anqing City, Anhui Province	110 m	OP034620
*L.insularis* (coastal area)			
01	Damao Island, Dinghai District, Zhoushan City, Zhejiang Province	22 m	OP034614
02	Chunxiao Town, Beilun District, Ningbo City, Zhejiang Province	8 m	OP034615
03	Shanjuan Town, Yixing City, Wuxi City, Jiangsu Province	42 m	ON611639
*L.chinensis*			
01	Bozhou Town, Xinhuang County, Huaihua City, Hunan Province	542 m	OP034613

### Acquisition, annotation, comparison and phylogenetic analysis of chloroplast genomes

DNA samples were extracted from leaves dried by silica gel according to the modified cetyltrimethylammonium bromide (mCTAB) extraction protocol (Doyle and Doyle 1987; Larridon et al. 2015). After Polymerase Chain Reaction (PCR), we used a NanoDrop 1000 Spectrophotometer and agarose gel electrophoresis to check the DNA quality. DNA library building and Genome Skimming and FastQC were outsourced to the Germplasm Bank of Wild Species in southwest China (China, Kunming), which employed Illumina HiSeq 6000 for analysis. The assembled 3G raw data from Genome Skimming have been analysed, using GetOrganelle v.1.7.1 to obtain the complete chloroplast genome, followed by genome annotation with PGA (Qu et al. 2019; Jin et al. 2020). This study was performed on the nine newly-reported complete chloroplast genomes (Table 1) and 14 complete chloroplast genomes of fertile diploid species were downloaded from NCBI. *Narcissuspoeticus* was selected as the outgroup (Könyves et al. 2018). See Fig. 3 for the specific accession number.

All sequences were aligned by MACSE v.2 (Ranwez et al. 2018). Maximum Likelihood (ML) and Bayesian Inference (BI) methods were used to determine the phylogenetic relationships, the best-fit model of DNA substitution being estimated by ModelFinder (Minh et al. 2013; Kalyaanamoorthy et al. 2017). ML analysis was conducted using the GTR + G + I model with 1000 bootstrap replicates by IQtree v.1.6.8 (Nguyen et al. 2015). Bayesian analysis was constructed with eight independent chains for 1,000,000 generations and sampling every 1000 generations by MrBayes v.3.2.6 (Ronquist et al. 2012; Zhang et al. 2022). All phylogenetic analyses were performed in Phylosuite (Zhang et al. 2020).

## Results

### Morphological comparison and karyotype observation

**Figure 1.**
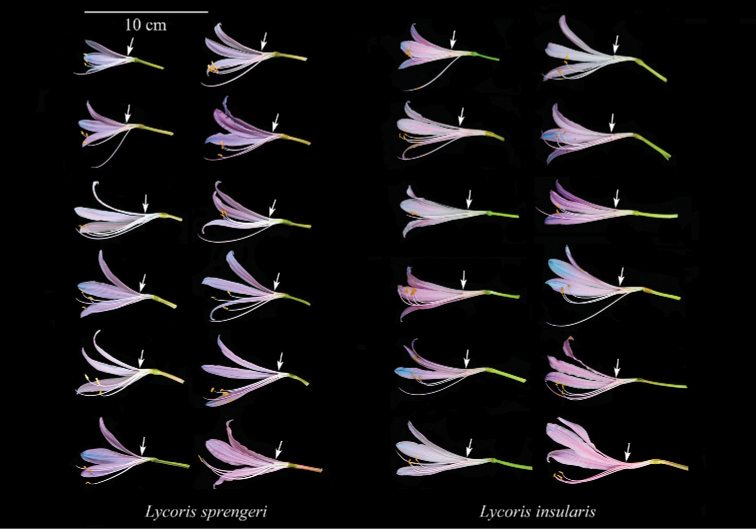
Anatomical comparison of *Lycorisinsularis* S.Y. Zhang & J.W.Shao, sp. nov. and *L.sprengeri* Comes ex Baker. The position indicated by the arrow is the top of the perianth tube.

**Figure 2.**
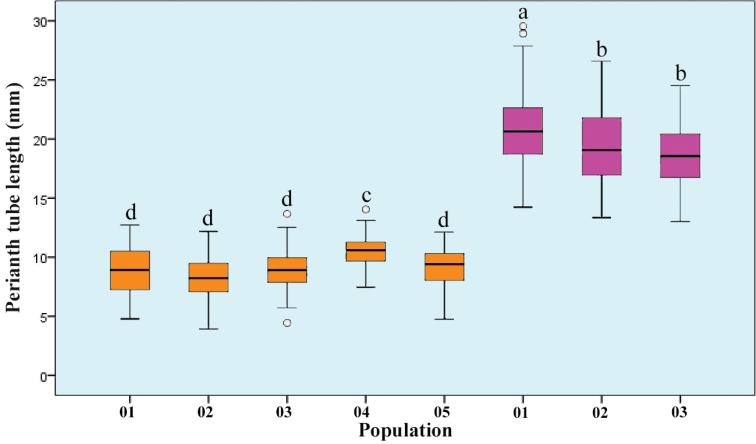
Comparison and variation of perianth tube length of *Lycorissprengeri* (01‒05, filled with orange) and *L.insularis* (01‒03, filled with magenta). In the boxplot, the horizontal line shows the median, the bottom and top of the box show the first and third quartiles. Boxplots marked with different letters differ significantly (post hoc test, P < 0.05).

**Figure 3.**
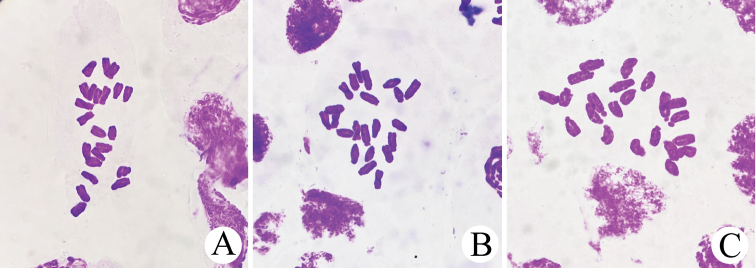
The karyotype of *Lycorisinsularis* S.Y. Zhang & J.W.Shao, sp. nov. **A** from the population 01 **B** from the population 02 **C** from the population 03. The information about populations is shown in Table 1.

*L.insularis* and *L.sprengeri* are very similar in many morphological characteristics, such as the leaves being slightly twisted when young, the apex being lavender and becoming a narrow and a long band when mature; the perianth is mainly pink, the apex is usually pale blue and the perianth lobes are rarely shrivelled, slightly or almost not rolling back. However, after careful observation, we found there are certain differences between them in the perianth tube length and flower colour. In all *L.sprengeri* populations observed so far, the perianth tube is short (0.5‒1.3 cm) and the flower colour ranged from nearly white (about 2%) to dark purple (about 2%), although most of the flowers are pale pink. In all populations of *L.insularis*, the perianth tube is relatively long (usually 1.5‒2.5 cm) and the flower colours are almost all either deep or shallow pink (Figs 1, 2). The chromosomes numbers of the three *Lycorisinsularis* sampled populations (Table 1) were consistently 22 (Fig. 3).

### Characteristics of the complete chloroplast genome

**Figure 4.**
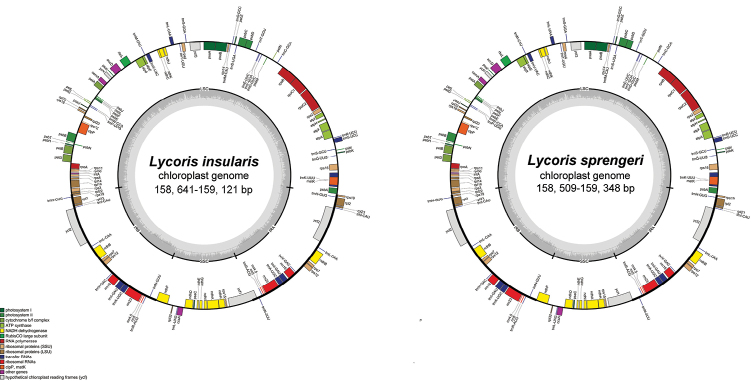
Plastid genome map of *Lycorisinsularis* S.Y. Zhang & J.W.Shao, sp. nov. and *L.sprengeri* Comes ex Baker.

The length of complete chloroplast genome of *Lycorisinsularis* comprised 158,641‒159,121 bp and *L.sprengeri* comprised 158,509‒159,348 bp (Fig. 4, Table 2). They both possessed typical quadripartite structure: IRa, IRb, LSC and SSC; the characteristics and statistics of the chloroplast genome are summarised in Table 2.

**Table 2.** Basic characteristics of chloroplast genomes of *Lycorisinsularis* and *L.sprengeri*.

Characteristic	*L.insularis*	*L.sprengeri*
Total length (bp)	158,641‒159,121	158,509‒159,348
GC%	37.7%‒37.8%	37.8%‒37.9%
LSC length (bp)	86,488‒86,590	86,483‒86,941
SSC length (bp)	18,496‒18,544	18,469‒18,501
IR length (bp)	26,806‒27,082	26,764‒27,018
Total genes	112	112
Protein-coding genes	78	78
rRNA genes	4	4
tRNA genes	30	30

### Molecular phylogenetic relationship

**Figure 5.**
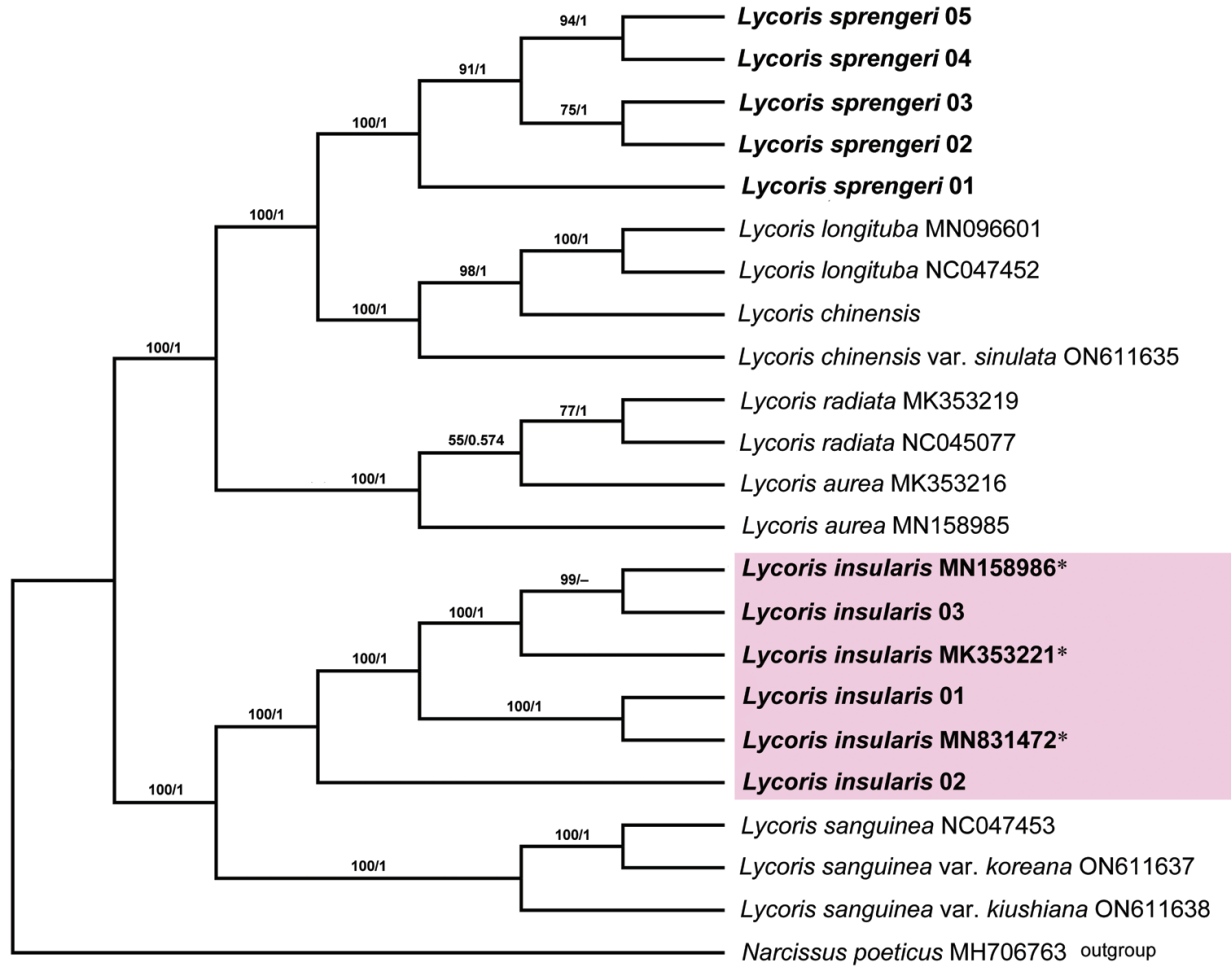
Phylogeny of the fertile diploid *Lycoris* species, based on the complete chloroplast genome. Numbers above branches are Maximum Likelihood bootstrap values (BS)/Bayesian posterior probability (BPP). The species name with accession numbers were download from NCBI. * showing those sequences which were misidentified as *L.sprengeri* in NCBI.

The phylogenetic relationship, based on the complete chloroplast genome containing eight species, is shown in Fig. 5. Five individuals, collected from the inland area (*L.sprengeri*) from different geographic populations, formed a monophyletic clade (bootstrap support (BS) = 100%), which is a sister clade to *L.longituba + L.chinensis*. Three individuals, collected from the coastal area (*L.insularis*) and three other sequences online (misidentified as *L.sprengeri*), composed a monophyletic clade (BS = 100%), which is a sister clade to *L.sanguinea*. *L.sprengeri* and *L.insularis*, respectively, are located in different clades and are separated at the base of the phylogenetic tree.

### Taxonomic treatment

#### Lycoris insularis

Taxon classificationPlantaeAsparagalesAmaryllidaceae

S.Y.Zhang & J.W.Shao sp. nov.

54F25751-0065-5C1C-B5AA-9029C1D3E62D

urn:lsid:ipni.org:names:77305347-1

Fig. 6

##### Type.

China. Zhejiang, Zhoushan City, Dinghai District, Damao Island, 29°56'55.4"N, 122°3'9.46"E, under the broad-leaved forest near the water on the Island, 22 m a.s.l., 18 August 2019, S.Y. Zhang, ZSY201908001 (holotype: ANUB008515!; isotypes: ANUB008516!, ANUB008517!, CSH0192378!, NPH001410!).

**Figure 6.**
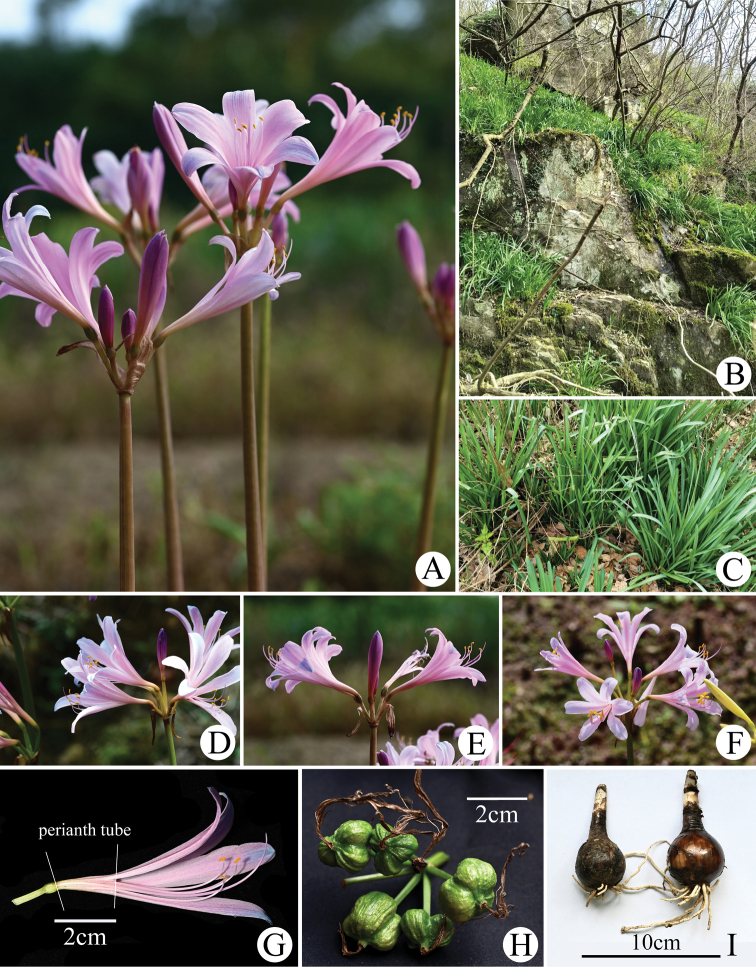
Morphology of *Lycorisinsularis* S.Y. Zhang & J.W.Shao, sp. nov. **A** inflorescence **B** habitat **C** plants in leaf stage **D, E, F** different individuals **G** anatomy of the flower **H** fruit **I** bulbs **G** was photographed by Cheng-sheng Li.

##### Diagnosis.

The new species resembles *Lycorissprengeri* by young leaves swirling and rising, tepals usually pink with blue apex and tepals length substantially similar, but differs in significantly longer perianth tube (1.5‒2.5 cm vs. 0.5‒1.3 cm).

##### Description.

Perennial herb. Bulb subglobose or ovate, 3‒5 cm in diameter, covered brown epidermis, with fine lines on the epidermis. Leaves linear, often 6‒9, blunt apex, appearing in winter or early spring, 40‒60 cm long, 6‒15 mm wide, pale green, mid-rib slightly sunken, covered with a little white powder. Inflorescence scapose, 40‒60 cm high, green or reddish-brown; two spathe bracts, lanceolate, about 3 cm long, 8‒12 mm wide; 5‒7 flowers per umbels, pedicels 1.5‒3 cm long, diameter about 3 mm; flowers usually pink with blue apex, occasionally white, blue or all pink; perianth lobes oblanceolate, 4.5‒7 cm long, about 0.9‒1.5 cm wide, apex slightly reversed; perianth tubes cream or pink, about 15‒25 mm long. Filaments 5‒7 cm long, pink, slightly longer than tepals; anther yellow, 3‒5 mm long; pistil length 6‒10 cm, pink or with purple apex. Ovary 5 mm in diameter, spherical and green. Capsules three-lobed, green or with light brown when mature; seeds black, spherical, about 7 mm in diameter.

##### Phenology.

Flowering from late July to late September; fruiting in September to October; leaves growing in winter or early spring (December to February of the following year).

##### Distribution and habitat.

**Figure 7.**
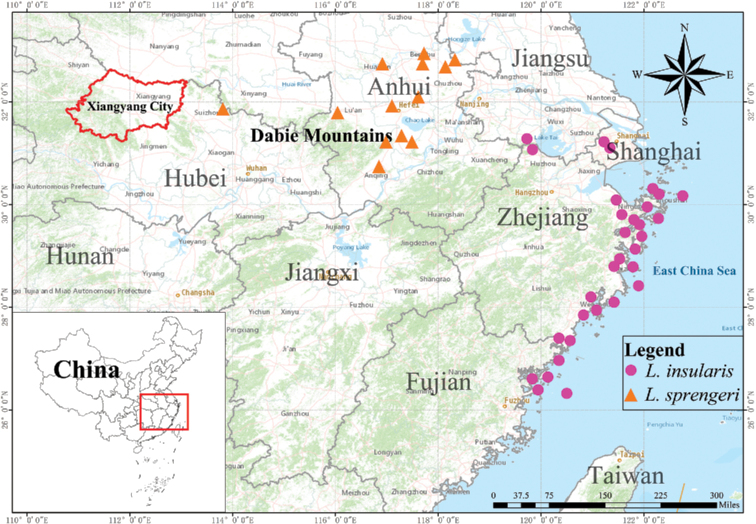
Distribution map of *Lycorisinsularis* S.Y. Zhang & J.W.Shao, sp. nov. and *L.sprengeri* Comes ex Baker. Distribution information is based on specimen inspections and actual surveys. Xiangyang City is the origin of the type specimen of *L.sprengeri*.

Most of the populations grow on the hillsides or island slopes near the sea (the coastal areas of Shanghai City, Zhejiang Province and Fujian Province) and sporadic populations grow around the inland hills and valleys (Fig. 7).

##### Chinese Name.

Hăi bīn shí suàn (海滨石蒜).

##### Etymology.

Latin insula, island, and -aris, belonging to; the specific epithet alludes to occurrence of the new species in Damao Island.

##### Reproduction.

This species can reproduce asexually by duplication of bulbs (about three times per two years) and also can sexually reproduce through seeds, taking about five years from seed germination to flowering.

##### Conservation status.

Compared with other species in *Lycoris*, *L.insularis* has a wider distribution area and many populations grow on inaccessible islands. After our field investigation, a large number of bulbs in Yixing and Huzhou were excavated for greening and landscaping, but there are still a large number of *L.insularis* populations in the wild in other areas. Thus, we classified its conservation status as LC (Least Concern), according to the IUCN Red List Criteria (IUCN 2019).

##### Additional specimen examined (paratypes).

China. Jiangsu Province: Yixing City, Shanjuan Cave, 25 Aug 1960, *Wen-zhe Fang* 00110645 (PE); Shanghai City: Songjiang District, Tianma Mountain, 8 Sept 1963, *Guang-jin Fan* 00110641 (PE); Songjiang District, Tianma Mountain, alt. 59 m, 14 Sept 2013, *Ting Zhang* 1393054 (KUN); Songjiang District, Dongshe Mountain, alt. 21 m, 28 Jul 2015, *Bin-jie Ge*, *Tian Li* CSH0098245 (CSH); Zhejiang Province: Putuo District, Taohua Island, alt. 19 m, 18 Sept 2017, *Yong-jie Guo*, *Li Huang*, *Zheng-yu Zuo & Ting Guo* 1446199 (KUN); Jiaojiang County, Jiangshan Island, alt. 3 m, 25 Oct 2016, *Yong-jie Guo*, *Qiao-rong Zhang*, *Li Huang*, *Lian-yi Li*, *Pei Li*, *De-ming He*, *Ying-hong Yang* 1451350 (KUN); Ruian County, Shuangfengshan Island, alt. 46 m, 11 Oct 2017, Y*ong-jie Guo*, *Qiao-rong Zhang*, *Yun-hua Fang*, *Xing-xu Sun* 1450112 (KUN); Putuo District, Putuo Mountain, alt. 62 m, 24 Aug 2015, *Bao-cheng Wu* NAS00591943, NAS00591944, NAS00591945 (NAS); Putuo District, Xiaomayi Island, 26 Jul 2011 *Qi Tian*, *Zheng-wei Wang* CSH0116798 (CSH); Putuo District, Daqing Mountain, alt.18 m, 21 Sept 2015, *Bin-jie Ge, Xu Yuan* CSH0101312 (CSH); Daishan County, Yushan Village, 21 Sept 2012, Xi-yang Ye CSH0032460 (CSH).

### Key to the fertile diploid native species of *Lycoris*

**Table:** 

1	Leaves appear in autumn (Sept‒Oct)	**2**
–	Leaves appear in winter or spring (Dec‒Feb)	**5**
2	Leaves apex obtuse	**3**
–	Leaves apex acuminate	**4**
3	Flowers red, stamen 6‒8 cm	***L.radiata***
–	Flowers rose-red, stamen 3‒3.5 cm	***L.wulingensis***
4	Pedicels 15‒22 mm, perianth tube length 1.2‒1.5 cm	***L.aurea***
–	Pedicels 8‒9 mm, perianth tube length 1.5‒2 cm	***L.traubii***
5	Leaves width 0.5‒1.5 cm, apex rose-red when young	**6**
–	Leaves width 1.5‒3 cm, apex always green	**8**
6	Flowers orange	***L.sanguinea***
–	Flowers pink, apex usually blue	**7**
7	Perianth tube length 1.5‒2.5 cm	***L.insularis***
–	Perianth tube length 0.5‒1.3 cm	***L.sprengeri***
8	Flowers actinomorphic, perianth tubes 3‒6 cm	***L.longituba***
–	Flowers zygomorphous, perianth tubes 1.5‒2.5 cm	**9**
9	Leaves width ca. 1.5 cm, tepals orange, apex usually red	***L.tsinlingensis***
–	Leaves width 1.5‒2.5 cm, tepals yellow to light orange	***L.chinensis***

## Discussion

Based on our research, especially the phylogenetic tree constructed by the complete chloroplast genome (Fig. 5), it can be clearly seen that those plants (identified as *L.sprengeri*) collected from the inland and coastal areas are two distinct entities (Comes and Baker 1902; Traub and Moldenke 1949). The original description and type specimen photos of *L.sprengeri* (in Kew, *Sprenger* K000901061, http://specimens.kew.org/herbarium/K000901061) both show that it has a short perianth tube (about 0.5 cm), which is consistent with those plants from inland populations collected by the authors. Therefore, we recognised that those inland plants with a short perianth tube (usually less than 1.3 cm) was the genuine *L.sprengeri*, while those coastal plants with a relatively long perianth tube (usually longer than 1.5 cm) is the new species, i.e. *L.insularis*.

The known karyotypes of fertile diploid species in *Lycoris* can be roughly divided into two categories: 2n = 12‒16 (such as *L.traubii*, *L.aurea*, *L.chinensis* and *L.longituba*) or 2n = 22 (such as *L.sanguinea*, *L.radiata*, *L.wulingensis* and *L.sprengeri*) (Kurita 1986; Hsu et al. 1994; Zhang et al. 2021; Zhang et al. 2022). Other sterile hybrid species are always with odd or allotriploid karyotypes. The chromosome number of *L.insularis* (2n = 22) is in line with the premise of being a fertile species. Furthermore, *L.insularis* has a relatively wide distribution area; its wild population can normally seed; and the seeds can develop into new plants at a high rate; thus, it can easily form a large population (more than 5,000 bulbs) and the individuals are scattered (i.e. do not show obvious clustering). All these factors indicate that *L.insularis* is a diploid fertile species and possibly has great value in the breeding and application of *Lycoris*.

## Supplementary Material

XML Treatment for Lycoris insularis
